# Inadequate pediatric reference ranges impede the diagnosis of X-linked hypophosphatemia and hypophosphatasia in Austria

**DOI:** 10.1007/s00508-025-02546-2

**Published:** 2025-05-19

**Authors:** Jojo Steininger, Magdalena Jablonska, Susanne Sagmeister, Gabriel Mindler, Adalbert Raimann

**Affiliations:** 1 Department of Pediatric and Adolescent Medicine, Division of Pediatric Pulmonology, Allergology and Endocrinology, Medical University of Vienna, Waehringer Guertel 18–20, 1090 Vienna, Austria; 2 Vienna Bone and Growth Center, Vienna, Austria; 3 Department of Pediatric Orthopedics, Orthopedic Hospital Speising, Vienna, Austria

**Keywords:** Pediatrics, Laboratory diagnostics, Rare bone diseases, Diagnostic errors, Health policy

## Abstract

**Background:**

Accurate diagnosis of many pediatric disorders relies on age-specific laboratory reference ranges. This is particularly important for rare disorders such as X‑linked hypophosphatemia (XLH) and hypophosphatasia (HPP), which present with decreases in serum phosphate and alkaline phosphatase (ALP), respectively.

**Objective:**

This study evaluated the use of pediatric reference ranges among Austrian medical laboratories.

**Methods:**

A comprehensive list of all extramural clinical laboratories was compiled. A standardized serum sample with pathological values for a 4-year-old child was dispatched to 26 extramural laboratories. The returned absolute and stated reference values were assessed and analyzed.

**Results:**

Of 22 responding laboratories, 18.2% used appropriate pediatric reference ranges for serum phosphate and 40.9% for ALP. In total, 54.5 and 36.4% of laboratories identified the sample as normal for serum phosphate or ALP, respectively.

**Conclusion:**

Despite accurate absolute value measurements, the majority of laboratories failed to identify the test sample as pathologic. In a real-world setting, the results obtained could lead to significant diagnostic delays and missed diagnoses in pediatric patients. The lack of regulatory requirements for pediatric-specific reference ranges in Austria and most European countries contributes to this problem. The study highlights the need for standardization and mandatory implementation of pediatric reference ranges to improve diagnostic accuracy for rare pediatric disorders across Europe.

## Introduction

The accurate diagnosis and management of pediatric conditions rely heavily on age-specific reference ranges for laboratory parameters. Unlike the stable, narrow ranges in adulthood, pediatric reference ranges show significant variability throughout childhood, reflecting complex physiological processes of growth and development [1, 2]. Without appropriate age-specific ranges, there is a substantial risk of misinterpreting results, leading to missed diagnoses or unnecessary interventions.

For rare disorders like X‑linked hypophosphatemia (XLH, OMIM 307800) and hypophosphatasia (HPP, OMIM 241510), age-appropriate reference ranges are crucial. These conditions, characterized by abnormal serum phosphate and alkaline phosphatase (ALP) levels, respectively, can be overlooked if adult ranges are applied to pediatric samples. In Austria and many other countries, there is no legal mandate for laboratories to use pediatric ranges or disclose their reference values, exacerbating diagnostic challenges. Despite their importance, many laboratories fail to implement pediatric-specific standards, even with accurate absolute value measurements. This can result in delayed diagnoses and missed opportunities for early intervention in rare disorders where timely diagnosis is critical, yet diagnostic delay is common [3, 4]. This study aimed to assess the use of pediatric reference ranges among Austrian medical laboratories.

## Methods

In the absence of publicly accessible data on extramural medical laboratories, a comprehensive web search including recommendation list from health insurances has been performed [5, 6]. A total of 115 laboratories providing clinical services in an extramural setting were identified. Sufficient contact data to dispatch samples was available for 26 individual laboratories. A standardized sample from an adult healthy individual with pathologic values for children (ALP 65 IU/mL, phosphate 1.08 mmol/L) was dispatched to all providers across Austria. Laboratories were instructed to analyze total ALP and serum phosphate, and interpret the results for a 4-year-old girl. Accuracy of absolute value measurements, stated reference ranges and diagnostic interpretation have been analyzed descriptively and compared to available reference data [7]. llama3.1:405b was used for language improvement [8].

## Results

In total, 22/26 laboratories (84.6%) completed the analysis and reported results. The findings revealed significant discrepancies in the interpretation and reference range utilization among the participating laboratories. Absolute accuracy of value determination was high with low variability between the different institutes (mean ALP: 66.8 ± 2.6 IU/mL; mean serum phosphate 1.18 ± 0.18 mmol/L).

**Fig. 1:**
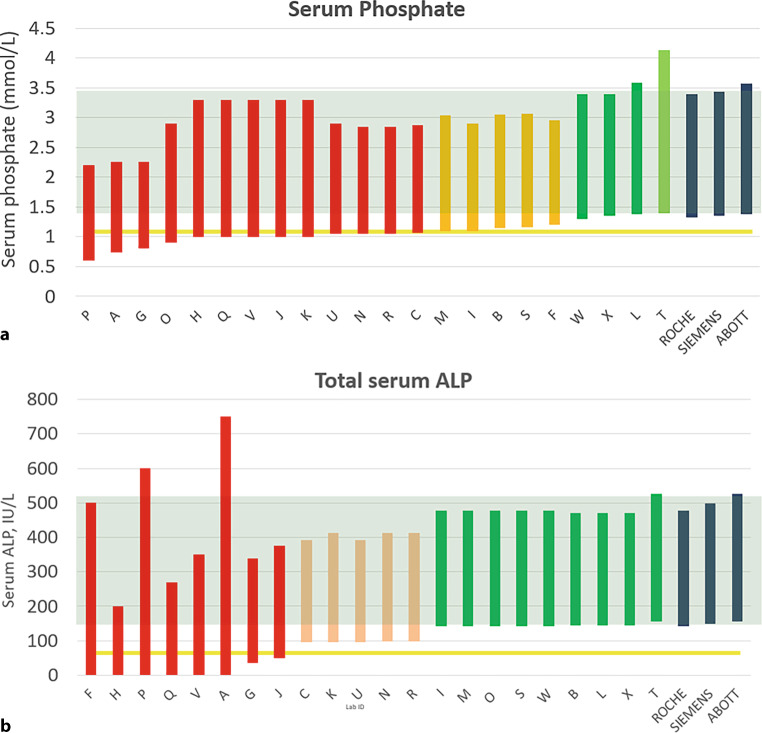
**a,** **b** Reference ranges provided for a 4-year-old girl by officially licensed medical laboratories. Institutions appear pseudonymized by letter code, colors from green to red indicate the deviation from official standards. CALIPER reference data for three commonly used lab systems are depicted for reference as blue bars on the right. Approximate overall standard reference range appears as a faint green background. The value of the standard sample has been marked as a yellow line. *ALP* alkaline phosphatase

Regarding serum phosphate levels, four laboratories (18.2%) provided reference values that approximated recommended pediatric ranges [1, 7]. The majority of institutes (12/22. 54.5%) interpreted the test sample’s results as normal, despite the values indicating pathologic hypophosphatemia in all available pediatric reference standards (Fig. 1b).

The interpretation of total ALP levels demonstrated similar inconsistencies. Less than half of the responding institutes (9/26, 40.9%) utilized appropriate pediatric reference values for total ALP. Eight laboratories (36.4%) reported ALP levels as within the normal range, even though they were clearly abnormal for a pediatric sample. Notably, six institutes failed to state any lower limit of normal for ALP, further complicating the interpretation of potentially low values indicative of conditions like HPP (Fig. 1a).

## Discussion

Laboratory medicine plays a crucial role in pediatric diagnostics. While clinicians rely on laboratory results, the interpretation provided by laboratories significantly influences clinical judgments. The widespread use of adult reference ranges for pediatric samples poses a substantial risk of misdiagnosis, particularly for conditions like XLH and HPP that rely on early detection of laboratory deviations [9, 10].

The observation that over half of Austrian laboratories misinterpreted pathological phosphate levels as normal is alarming and should be viewed as a transnational issue. Due to this study characteristics, data on the used assay could not be acquired systematically. Variations of reference ranges vary between different manufacturers as shown in the CALIPER database for most commonly used systems (Lower limits of normal: ALP 130–160 IU/L; serum phosphate 1.33–1.57 mmol/L. Upper limits of normal: ALP 315–417 U/L; serum phosphate: 2.09–2.33 mmol/L) [7]. The narrow range of absolute values retrieved by this study suggests a low impact of interassay variability, and primarily insufficiencies in the postanalytical phase, specifically in result interpretation and reporting [10].

The absence of legal requirements for pediatric-specific reference ranges in Austria and most European countries reflects a broader regulatory gap. This leaves correct interpretation of pediatric lab values to the discretion of clinical laboratories, associated with implementation costs. The implications extend beyond XLH and HPP, potentially impacting the diagnosis and management of various pediatric disorders, including immunodeficiencies and nephrologic and hematologic conditions. Consequences may include diagnostic delays, complications, and additional healthcare costs, though data is limited.

Study limitations include potential missed laboratories due to lack of public lists, data limited to extramural institutes, interassay variability and reference values collected only for the specified age and sex of the sample. However, the results indicate a need for enhanced collaboration between laboratory medicine and clinicians.

A three-step approach is suggested to improve laboratory assessment quality:

1. Raise awareness in laboratory medicine societies and federal health legislation to develop national recommendations for pediatric-specific reference ranges.
2. Link public health insurance reimbursement to quality standards for pediatric sample reference ranges.
3. Implement a publicly available white-list of institutes for critical parameters, including standardized pediatric reference ranges, at national and European levels.

## Conclusion

This study reveals a significant and widespread deficiency in the use of pediatric reference ranges among Austrian medical laboratories. While single laboratories provide excellent standards, the high rates of misinterpretation in the screening values for X‑linked hypophosphatemia (XLH) and hypophosphatasia (HPP) underscore the urgent need for standardization and mandatory implementation of pediatric reference ranges in medical laboratories across Austria and, by extension, the European Union.
